# Chondroitin polymerizing factor promotes development and progression of colorectal cancer via facilitating transcription of VEGFB

**DOI:** 10.1111/jcmm.18268

**Published:** 2024-05-22

**Authors:** Yuan Huang, Zhen Zhang, Hanxing Tong, Wenzheng Qin, Quanlin Li, Lili Ma, Zhong Ren, Weifeng Chen, Yiqun Zhang, Yunshi Zhong, Liqing Yao, Pinghong Zhou

**Affiliations:** ^1^ Shanghai Collaborative Innovation Center of Endoscopy, Endoscopy Center and Endoscopy Research Institute, Zhongshan Hospital Fudan University Shanghai China; ^2^ Department of General Surgery, Zhongshan Hospital Fudan University Shanghai China

**Keywords:** CHPF, colorectal cancer, molecular mechanism, tumour promotor, VEGFB

## Abstract

Colorectal cancer (CRC) is a highly prevalent malignancy affecting the digestive system on a global scale. This study aimed to explore the previously unexplored role of CHPF in the progression of CRC. Our results revealed a significant upregulation of CHPF expression in CRC tumour tissues compared to normal tissues, with its levels correlating with tumour malignancy. In vitro experiments using CRC cell lines demonstrated that inhibiting CHPF expression suppressed cell proliferation, colony formation and cell migration, while promoting apoptosis. Conversely, overexpressing CHPF had the opposite effect. Additionally, our xenograft models in mice confirmed the inhibitory impact of CHPF knockdown on CRC progression using various cell models. Mechanistic investigations unveiled that CHPF may enhance VEGFB expression through E2F1‐mediated transcription. Functionally, suppressing VEGFB expression successfully mitigated the oncogenic effects induced by CHPF overexpression. Collectively, these findings suggest that CHPF may act as a tumour promoter in CRC, operating in a VEGFB‐dependent manner and could be a potential target for therapeutic interventions in CRC treatment.

## 1 INTRODUCTION

Colorectal cancer (CRC) is a malignant neoplasm arising in the colon or rectum.^1^, ^2^ Colorectal cancer (CRC) is currently a highly prevalent malignancy globally, with its incidence steadily rising over the years. This escalating trend underscores the significant threat CRC poses to human health.^3^ According to the latest cancer statistics in United States, the incidence of CRC in men and women accounted for 9% and 7% of the total morbidity of male and female tumours, respectively, and ranked third among male and female cancers.^4^, ^5^ Currently, the primary approach for managing CRC involves surgical resection as the cornerstone of treatment, complemented by adjuvant modalities such as chemotherapy, radiation therapy and immunotherapy.^6^, ^7^ However, because of the recurrence and metastasis following surgical resection, and the low efficacy as well as serious side‐effects of adjuvant treatment, the prognosis of CRC remains poor and unsatisfactory.^8^, ^9^, ^10^, ^11^ Recently, through targeting the specific targets of tumour cells, molecular targeted therapy exhibits stronger anti‐tumour activity while reducing side effects on normal cells, paving a novel path for the treatment of CRC.^12^, ^13^ Consequently, comprehending the molecular mechanisms underlying CRC holds paramount importance and has garnered significant attention, serving as a fundamental basis for the advancement of molecular targeted therapies.

Chondroitin sulfate (CS), a sulfated glycosaminoglycan, is primarily observed in the extracellular matrix of humans and other animal species, particularly within the connective tissue.^14^, ^15^ The crucial role of CS in the regulation of various diseases, encompassing osteoarthritis,^14^, ^16^ cardiovascular and cerebrovascular disorders,^17^ central nervous system pathologies^18^ and human cancers^19^ has been previously reported. CS has been implicated in the modulation of immune responses and the inhibition of angiogenesis, thereby exerting potential anticancer effects.^20^, ^21^ The biosynthesis of CS involves a complex cascade of events and relies on the activity of six glycosyltransferases. Within this group of enzymes, chondroitin polymerizing factor (CHPF) serves as an essential coenzyme for the synthesis of double disaccharide units in chondroitin sulfate (CS), working in conjunction with human chondroitin synthase.^15^, ^22^ Furthermore, aside from its involvement in CS biosynthesis, CHPF has been associated with diverse human cancers.^23^, ^24^ For example, Fan et al. demonstrated a significant inhibition of cell proliferation and promotion of apoptosis upon downregulation of CHPF expression in glioma cells, potentially linked to G0/G1 cell cycle arrest.^25^ Nevertheless, the role of CHPF in colorectal cancer (CRC) remains undisclosed and unexplored.

In this investigation, we have identified CHPF as a promoter of colorectal cancer (CRC) progression, thus suggesting its potential as a therapeutic target for CRC treatment. CHPF demonstrated upregulation in CRC tumour tissues, and its increased expression was associated with higher tumour malignancy grades. In vitro experiments elucidated that manipulating CHPF expression, either through knockdown or overexpression, could respectively hinder or enhance CRC development by modulating cellular proliferation, apoptosis and migration. The inhibitory impact of CHPF knockdown on CRC was further validated through in vivo experiments utilizing mouse xenograft models. Furthermore, we provide evidence that CHPF may promote CRC by directly regulating VEGFB, as demonstrated by the significant attenuation of CHPF's overexpression effects on CRC upon VEGFB knockdown.

## 2 MATERIALS AND METHODS

### 2.1 Cell culture

Human colon cancer cell lines HCT116, RKO and Caco2 were purchased from BeNa Technology (Hangzhou, Zhejiang, China). Human colorectal adenocarcinoma cell line Caco2 HCT116 and RKO cell lines were cultured in RPMI‐1640 medium (Gibco, Rockville, MD, USA) containing 10% FBS (Gibco, Rockville, MD, USA). Caco2 cells were grown in a 90% DMEM medium (Gibco, Rockville, MD, USA) supplemented with 10% FBS additive. All cells were humid cultured in a 37°C 5% CO_2_ incubator.

A lentivirus solution containing a titre of 1 × 10^8^ TU/mL and specifically targeting the desired molecules was used to infect logarithmic growth phase CRC cells at a density of 2 × 10^5^ cells/well. The cells were then cultured for a duration of 72 h. Microscopic fluorescence analysis was employed to assess the efficiency of cell infection.

The treatment of chABC (Sigma, St. Louis, MO, USA) was performed at 0.1 U/mL for cell proliferation assay and 0.5 U/mL for colony formation assay, respectively.

### 2.2 Immunohistochemistry analysis

Colon cancer and normal tissue microarrays were acquired from Shanghai Outdo Biotech Co., Ltd. (#XT17‐024, Shanghai, China). A total of 101 tissue samples, comprising 180 spots, were collected between July 2006 and May 2007 for immunohistochemistry analysis. Prior to the surgical operation, patients were provided with informed consent and the experimental design received approval from the Institutional Animal Care and Use Committee of Fudan University. The tissue specimens were dewaxed using xylene and then rehydrated with ethanol. Subsequently, a primary antibody was applied and incubated overnight at 4°C. A secondary antibody was utilized for an additional 2 h incubation at room temperature. Finally, diaminobenzidine staining was performed on the tissue samples. Microscopic images of the spots were captured and analysed using CaseViewer_2.0 and ImageScope_v11 software. Based on the sum of staining intensity and staining extent scores, the specimens were categorized as negative (0), positive (1–4), ++ positive (5–8) or +++ positive (9–12). Antibodies used are shown in Table S1.

### 2.3 Plasmid construction, lentivirus infection and transfection

The full‐length human complementary DNA of CHPF/E2F1 was amplified through PCR and subsequently cloned into the BR‐V112 vector obtained from Shanghai Yibeirui Biomedical Science and Technology Co., Ltd. For knockdown purposes, specific RNA sequences targeting CHPF (5′‐AGCTGGCCATGCTACTCTTTG‐3′) and VEGFB (5′‐AGGAAAGTGGTGTCATGGATA‐3′, 5′‐CAGTGTGAATGCAGACCTAAA‐3′, 5′‐AGCACCAAGTCCGGATGCAGA‐3′) were designed by Shanghai Yibeirui Biomedical Science and Technology Co., Ltd. These RNA sequences were inserted into the BR‐V‐108 vector, and the expression vectors were confirmed through DNA sequencing analysis.

The EndoFree maxi plasmid kit from Tiangen (Beijing, China) was employed for plasmid extraction. Transfection of the shRNA expression vector and packaging vector into 293 T cells was achieved using Lipofectamine 2000 transfection reagent obtained from Thermo Fisher Scientific (Waltham, MA, USA). Following cell culture, lentivirus was collected for subsequent cell transduction experiments.

### 2.4 RNA extraction and RT‐qPCR

HCT116, RKO and Caco2 cells infected with lentivirus for 72 h were harvested and TRIzol reagent (Sigma, St. Louis, MO, USA) was added for effective cell lysis and nucleoprotein dissociation. Chloroform was subsequently added for phase separation, followed by RNA precipitation using isopropanol. The RNA pellet was washed, resuspended and quantified. Then, the quality of total RNA was evaluated by Nanodrop 2000C spectrophotometer (Thermo Fisher Scientific, Waltham, MA, USA) according to the manufacturer's instructions. Two micrograms of total RNA was reverse transcribed using Promega M‐MLV Kit (Promega, Heidelberg, Germany) and quantitative real‐time PCR was performed with SYBR Green mastermix Kit (Vazyme, Nanjing, Jiangsu, China) and applied Biosystems 7500 Sequence Detection system. GAPDH was used as inner control, and the related primers used for the PCR reaction are shown in Table S2. The relative quantitative analysis in gene expression data was analysed by the 2^−ΔΔCt^ method.

### 2.5 Western blotting assay

HCT116, RKO and Caco2 cells were lysed in ice‐cold RIPA buffer (Millipore, Temecula, CA, USA) and the total protein concentration was detected by BCA Protein Assay Kit (HyClone‐Pierce, Logan, UT, USA). Twenty micrograms of proteins were separated by 10% SDS‐PAGE (Invitrogen, Carlsbad, CA, USA) and were transferred onto PVDF membranes. Then the membranes were incubated with antibodies which were detailed in Table S1. The blots were visualized by enhanced chemiluminescence (ECL) (Amersham, Chicago, IL, USA).

### 2.6 Celigo cell counting assay

Caco2 cells were collected after transfection for 72 h and seeded into 96‐well plates with a cell density of 3000 cells per well. Cells were further cultured in RPMI‐1640 medium containing 10% FBS at 37°C with 5% CO_2_ for 120 h. Cell counting was accomplished by Celigo image cytometer (Nexcelom Bioscience, Lawrence, MA, USA) on days 1, 2, 3, 4 and 5 and the cell proliferation curve was drawn.

### 2.7 MTT assay

HCT116 and RKO cells transfected with lentivirus were seeded into a 96‐well plate at a density of 3000 cells per well in triplicate. Following cell seeding, 20 μL of MTT solution (5 mg/mL, GenView, El Monte, CA, USA) was added and incubated for 4 h. Subsequently, 100 μL of DMSO solution was added to each well. The absorbance values at 490 nm were measured using a microplate reader (Tecan, Männedorf, Zürich, Switzerland), with a reference wavelength of 570 nm. The cell viability ratio was calculated using the following equation: Cell viability (%) = (optical density (OD) treated/OD control) × 100%.

### 2.8 Flow cytometry for apoptosis

HCT116, RKO and Caco2 cells transfected with lentivirus were seeded in triplicate in 6‐well plates with 2 mL of medium per well, at a density of 1 × 10^3^ cells/mL. The cells were then cultured for 5 days. Floating cells were collected and washed with ice‐cold D‐Hanks at 4°C, followed by trypsinization. After centrifugation at 1000 × g, the cells were resuspended in binding buffer. Staining was performed by adding 5 μL of Annexin V‐APC (eBioscience, San Diego, CA, USA) and 5 μL of propidium iodide (Sigma, St Louis, MO, USA) to the cell suspension, avoiding exposure to light. Apoptosis analysis was conducted using FACSCalibur (BD Biosciences, San Jose, CA, USA).

### 2.9 Wound healing assay

HCT116, RKO and Caco2 cells transfected with lentivirus were seeded at a density of 5 × 10^4^ cells/well in a 96‐well dish for cell culture. A 96‐wounding replicator (VP scientific, San Diego, CA, USA) was used to create scratches across the cell layer when it reached 90% confluence. RPMI‐1640 medium supplemented with 0.5% FBS was added for further culturing. Fluorescence microscope images were captured at 0 h, 16 h and 24 h after scratching. The cell migration rates for each group were then calculated.

### 2.10 Colony formation assay

HCT116, RKO and Caco2 cells in the logarithmic growth phase were trypsinized, resuspended and seeded into six‐well plates in triplicate with a density of either 500 or 1000 cells per well. The cells were cultured in RPMI‐1640 medium supplemented with 10% fetal bovine serum, and the culture medium was refreshed every 72 h. Fluorescence microscope images of cell clones were captured using an Olympus microscope (Tokyo, Japan). To fix the cells, 1 mL of 4% paraformaldehyde was added, followed by staining with 500 μL of Giemsa. The cells were then washed, dried and photographed using a digital camera. The colony forming rate was calculated as the percentage of colonies formed relative to the number of cells initially seeded, using the formula: (colony number/inoculated cell number) × 100%.

### 2.11 Transwell assay

The Transwell assay was conducted using the Corning Transwell Kit (Corning, NT, USA). HCT116, RKO and Caco2 cells successfully infected with lentivirus were seeded in the upper chamber of a 24‐well plate at a density of 1 × 10^5^ cells/well, with 100 μL of medium without FBS. The lower chamber was filled with 500 or 600 μL of medium supplemented with 30% FBS. The cells were incubated at 37°C with 5% CO_2_ for 24–72 h. After incubation, the cells were fixed with 4% formaldehyde and stained with Giemsa to assess their migration ability. The migration analysis was performed by examining the cells that migrated to the lower chamber. The experiment was repeated in triplicate using three separate wells.

### 2.12 Human apoptosis antibody array

The detection of related genes in the human apoptosis signalling pathway was conducted using the Human Apoptosis Antibody Array (R&D Systems, Minneapolis, MN, USA) according to the manufacturer's instructions. Lentivirus‐transfected Caco2 cells were collected, washed and then lysed using a lysis buffer to extract total protein. The protein samples (0.5 mg/mL) were incubated with the blocked array antibody membrane overnight at 4°C. After washing, a 1:100 dilution of the Detection Antibody Cocktail was added and incubated for 1 h, followed by incubation with an HRP‐linked streptavidin conjugate for 1 h. All spots on the membrane were visualized using enhanced chemiluminescence (ECL) (Amersham, Chicago, IL, USA), and the signal densities were analysed using ImageJ software (National Institute of Health, Bethesda, MD, USA).

### 2.13 Chromatin immunoprecipitation (ChIP)‐qPCR

Chromatin immunoprecipitation (ChIP) was conducted using the Chromatin Extraction Kit (Abcam, Cambridge, MA, USA) and ChIP Kit Magnetic‐One Step (Abcam, Cambridge, MA, USA) following the manufacturer's instructions. Reverse transcription‐polymerase chain reaction (RT‐PCR) was performed using the aforementioned method.

### 2.14 Dual‐luciferase assay

The VEGFB promoters were cloned into the GL002 vector, and these plasmids were transfected into RKO cells with or without overexpression of E2F1. Following transfection, luciferase activity was measured using a Promega Dual‐Luciferases Reporter Assay kit, following the manufacturer's instructions. The relative Renilla luciferase activity was normalized to the firefly luciferase activity. For VEGFB‐MUT, a mutant luciferase plasmid with mutation in the 1840 bp–1855 bp segment of the 2 kb sequence of the promoter of the VEGFB was constructed. Herein, sequence gcgggaggcgggaggg was mutated to TACTTGTTGTTTCTTT.

### 2.15 Animal experiments and fluorescence imaging

All animal experiments were conducted in accordance with the guidelines and approved by the Institutional Animal Care and Use Committee of Fudan University. The study was carried out at the Animal Laboratory of Fudan University. Four‐week‐old nude mice (BALB/c) were obtained from Shanghai Lingchang Experimental Animals Co., Ltd (Shanghai, China). The mice were randomly divided into two groups: shCHPF group and shCtrl group. Subcutaneous injections of 4 × 10^6^ RKO cells were performed on each mouse to induce tumour formation. The mice's weight and tumour sizes were measured twice a week using a calliper, and the tumour volume was calculated using the formula *π*/6 × *L* × *W* ^2^ (*W* representing the width at the widest point and *L* representing the perpendicular width). Tumour burden was assessed weekly using the IVIS Spectrum Imaging System (Perkin Elmer, Waltham, MA, USA) for fluorescence imaging. After 29 days, the mice were sacrificed, and the tumours were extracted and imaged. Pentobarbital sodium was used as an anaesthetic during the animal experiments.

### 2.16 Ki‐67 immunostaining

Tumour tissues obtained from mice were utilized for Ki67 immunostaining. The tissues were fixed and embedded in formalin and paraffin, and 2 μm slides were prepared. Deparaffinization and rehydration of the slides were carried out by immersing them in xylene and 100% ethanol. Subsequently, all slides were blocked using PBS‐H_2_O_2_. Primary antibody Ki‐67 (1/200) was incubated with the slides overnight at 4°C, followed by incubation with 1:400 goat anti‐rabbit IgG H&L (HRP) (Abcam, Cambridge, MA, USA). Haematoxylin and Eosin (Baso, Zhuhai, Guangdong, China) staining was performed for HE staining. IHC staining was conducted using DAB substrate and haematoxylin. Finally, the stained slides were examined under a microscope.

### 2.17 Gene chip analysis

The gene expression profile analysis in RKO cells transfected with shCHPF or shCtrl was conducted by Shanghai Yibeirui Biomedical Science and Technology Co., Ltd. Total RNA was extracted from shCtrl and shCHPF RKO cells using the RNeasy kit (Sigma, St. Louis, MO, USA). The quality of total RNA was assessed using Agilent 2100 (Agilent, Santa Clara, CA, USA), and its quantity was determined using Nanodrop 2000 (Thermo Fisher Scientific, Waltham, MA, USA). RNA sequencing was performed using the human GeneChip primeview (Affymetrix, Santa Clara, CA, USA) according to the manufacturer's instructions, and the results were scanned using the Affymetrix Scanner 3000 (Affymetrix, Santa Clara, CA, USA). Statistical significance of the raw data was assessed using a Welch *t*‐test with Benjamin–Hochberg FDR (FDR <0.05 considered significant). Ingenuity Pathway Analysis (IPA) (Qiagen, Hilden, Germany) was conducted for all significantly differentially expressed genes.^26^ A |*Z*‐score| > 2 was considered to be indicative of meaningful differences.

### 2.18 Statistical analyses

The data were presented as mean ± SD, and statistical analysis was performed using Student's *t*‐test to determine the significance between the experimental group and control group. All statistical analyses were conducted using SPSS 17.0 (IBM, SPSS, Chicago, IL, USA), and a *p*‐value of <0.05 was considered statistically significant. The difference in CHPF gene expression was analysed using the Rank Sum test. Mann–Whitney *U* analysis and Spearman Rank correlation analysis were utilized to examine the relationship between CHPF expression and tumour characteristics in patients. Graphs were generated using GraphPad Prism 6.01 (Graphpad Software, La Jolla, CA, USA).

## 3 RESULTS

### 3.1 Levels of CHPF were upregulated in cases of colorectal cancer

**FIGURE 1:**
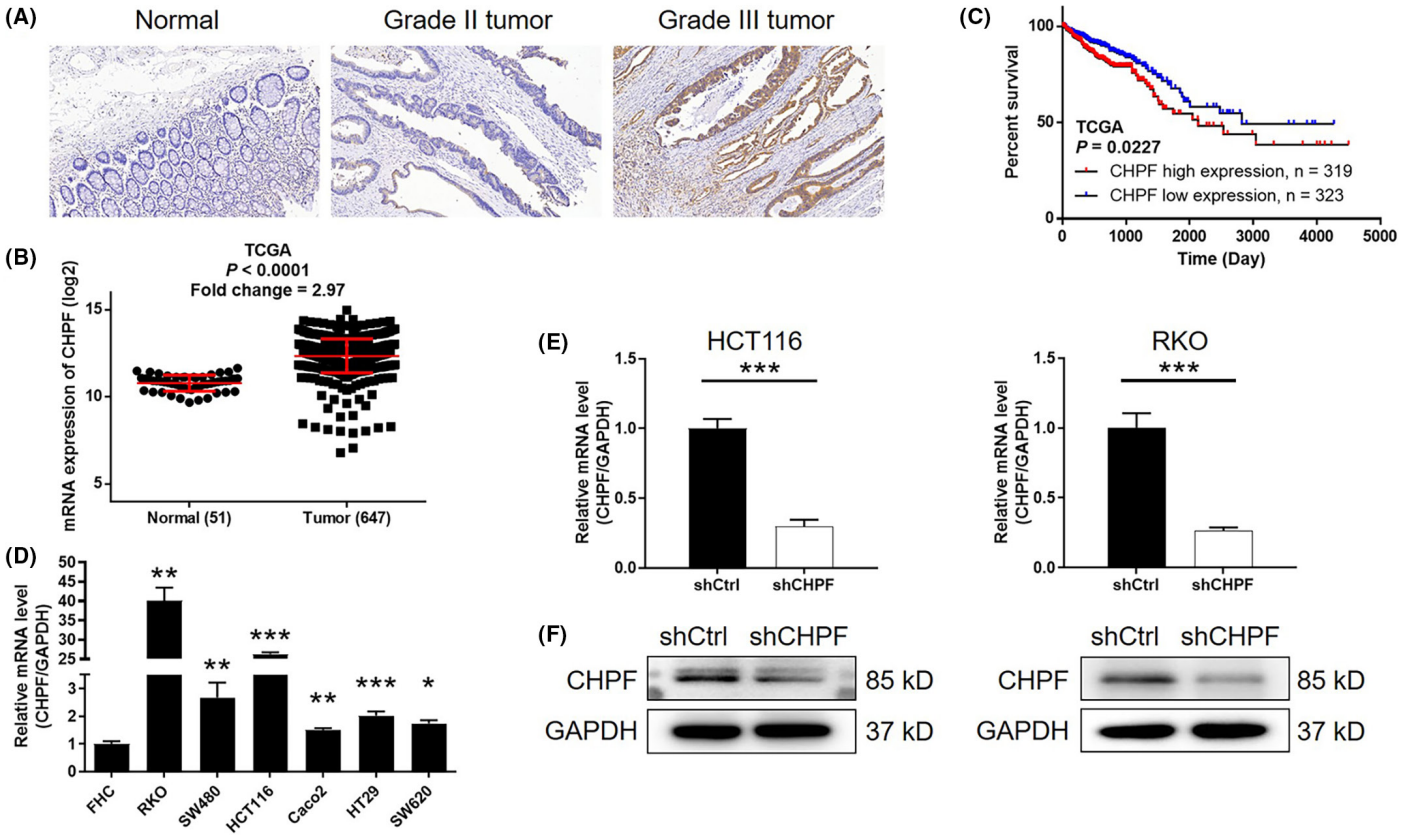
CHPF was upregulated in CRC. (A) The expression of CHPF in CRC tumour tissues and normal tissues was detected by IHC. (B) The expression profiling data collected from TCGA showed that CHPF was upregulated in CRC. (C) The prognostic data obtained from TCGA indicated that high CHPF expression was associated with poor prognosis. (D) The background expression of CHPF in normal human epithelial cell line FHC and CRC cell lines including HT‐29, SW480, SW620, RKO, HCT116 and Caco2 was detected by qPCR. (E) The efficiency of lentivirus‐mediated CHPF knockdown in HCT116 and RKO cells was evaluated by qPCR. (F) The knockdown of CHPF in HCT116 and RKO cells was verified by western blotting. The data were expressed as mean ± SD (*n* ≥ 3), *p < 0.05; **p < 0.01; ***p < 0.001.

To investigate the role of CHPF in colorectal cancer (CRC), immunohistochemistry (IHC) analysis was conducted on clinical specimens obtained from CRC patients to evaluate the expression levels of CHPF protein. The results revealed a significant elevation in CHPF expression in tumour tissues compared to normal tissues (*p* < 0.001, Figure 1A and Table 1). Moreover, statistical analysis demonstrated a substantial correlation between high CHPF expression levels and advanced malignant grade (*p* < 0.001, Table 2). Analysis of gene expression profiling data from The Cancer Genome Atlas (TCGA) further validated the upregulation of CHPF in CRC tumour tissues (*p* < 0.001, Figure 1B). Survival analysis indicated a significant and positive association between high CHPF expression levels and poor prognosis (*p* = 0.023, Figure 1C). Furthermore, the expression of CHPF was examined using quantitative polymerase chain reaction (qPCR) in the human colon epithelial cell line FHC and various CRC cell lines. The analysis revealed higher CHPF expression levels in CRC cell lines compared to FHC cells (Figure 1D). Lentiviral constructs expressing shCtrl or shCHPF were then transfected into HCT116 and RKO cells to establish cell models with CHPF knockdown. The transfection efficiency, determined by fluorescence signals, exceeded 80% in both cell lines (Figure S1). qPCR analysis confirmed a knockdown of CHPF expression by 70.63% and 73.83% in HCT116 and RKO cells, respectively (*p* < 0.01, Figure 1E). Western blotting supported these findings (Figure 1F). Collectively, these results demonstrate the upregulated expression of CHPF in CRC and the successful establishment of CHPF knockdown cell models for future research purposes.

**TABLE 1:** Expression patterns of CHPF in CRC tissues and normal tissues revealed in immunohistochemistry analysis.

CHPF expression	Tumour tissue		Normal tissue	
	Cases	Percentage	Cases	Percentage
Low	39	39.8%	77	100%
High	59	60.2%	0	–

*p* < 0.001.

**TABLE 2:** Relationship between CHPF expression and tumour characteristics in patients with CRC.

Features	No. of patients	CHPF expression		*p* Value
		Low	High	
All patients	92	36	56	
Age (years)				
<71	44	16	28	0.605
≥71	48	20	28	
Gender				
Male	54	21	33	0.768
Female	43	18	25	
Lymph node positive				
≤0	47	20	27	0.302
>0	38	12	26	
Tumour size				
<5.5	48	18	30	0.739
≥5.5	49	20	29	
Grade				
II	50	28	22	0.001
III	48	11	37	
AJCC stage				
1	5	2	3	0.295
2	53	23	30	
3	36	13	23	
4	3	0	3	
*T* infiltrate				
T1	1	0	1	0.135
T2	5	3	2	
T3	74	32	42	
T4	14	3	11	
Lymphatic metastasis (*N*)				
N0	58	25	33	0.239
N1	27	9	18	
N2	11	3	8	

*p* < 0.01.

### 3.2 The knockdown of CHPF inhibited CRC development in vitro

**FIGURE 2:**
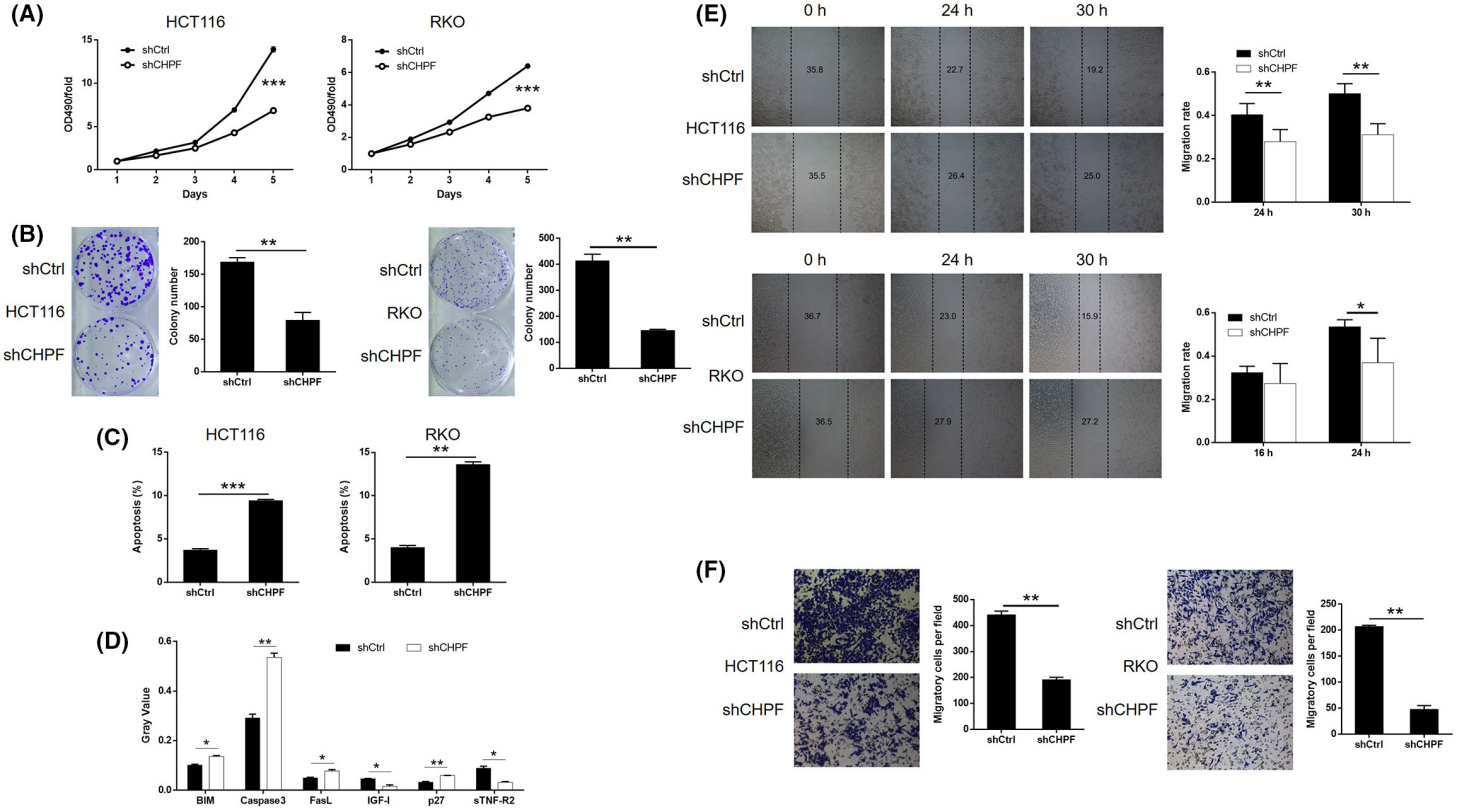
CHPF knockdown inhibited development of CRC in vitro. (A) MTT assay was performed to examine the effects of CHPF knockdown on cell proliferation of HCT116 and RKO cells. (B) The effects of CHPF knockdown on the ability of HCT116 and RKO cells to form colonies were evaluated by colony formation assay. (C) Flow cytometry was used to detect cell apoptosis of HCT116 and RKO cells with or without CHPF knockdown. (D) The differentially expressed apoptosis‐related proteins identified in RKO cells with or without CHPF knockdown by Human Apoptosis Antibody Array. (E, F) Cell migration ability of HCT116 and RKO cells with or without CHPF knockdown was detected by wound healing assay (E) and Transwell assay (F). The figures are representative data from at least three independent experiments. The data were expressed as mean ± SD (*n* ≥ 3), *p < 0.05; **p < 0.01; ***p < 0.001.

To investigate the role of CHPF in colorectal cancer (CRC), a series of in vitro experiments were conducted. MTT assays demonstrated a significant reduction in cell proliferation, with 51.82% and 45.93% decreases observed in HCT116 and RKO cells, respectively, following CHPF knockdown (*p* < 0.001, Figure 2A). Colony formation assays revealed a decline of 52.95% and 64.68% in colony numbers in the shCHPF group compared to the shCtrl group after 14 days of culture in both HCT116 and RKO cells, respectively (*p* < 0.01, Figure 2B). Flow cytometry analysis of cell apoptosis showed a 2.53‐fold and 3.38‐fold increase in the proportion of apoptotic cells in the shCHPF group compared to the shCtrl group in HCT116 and RKO cells, respectively, indicating the ability of CHPF knockdown to induce apoptosis (*p* < 0.01, Figure 2C). Furthermore, the impact of CHPF knockdown on apoptosis‐related proteins was examined using a Human Apoptosis Antibody Array in RKO cells with or without CHPF knockdown (Figure S2). The results revealed an upregulation of BIM, Caspase3, FasL and p27 expression levels, while IGF‐4 and sTNF‐R2 expression levels were downregulated upon CHPF knockdown (*p* < 0.05, Figure 2D). To assess the effect of CHPF knockdown on cell motility, wound healing and Transwell assays were performed. CHPF knockdown resulted in a reduction of 37.84% and 30.97% in cell migration rate in HCT116 and RKO cells, respectively (Figure 2E; *p* < 0.05). Similarly, Transwell assays showed a decrease of 56.59% in HCT116 cells and a 76.85% inhibition in RKO cells (*p* < 0.01, Figure 2F). These collective findings indicate that CHPF knockdown can inhibit the in vitro development of CRC. Moreover, it was demonstrated that the treatment of cells with chondrotinase (chABC, 0.5 U/mL) could also inhibit cell proliferation and colony formation (Figure S3), which suggested the potential function of chondroitin sulfate in colorectal cancer development.

### 3.3 The knockdown of CHPF inhibited the in vivo growth of CRC tumours

**FIGURE 3:**
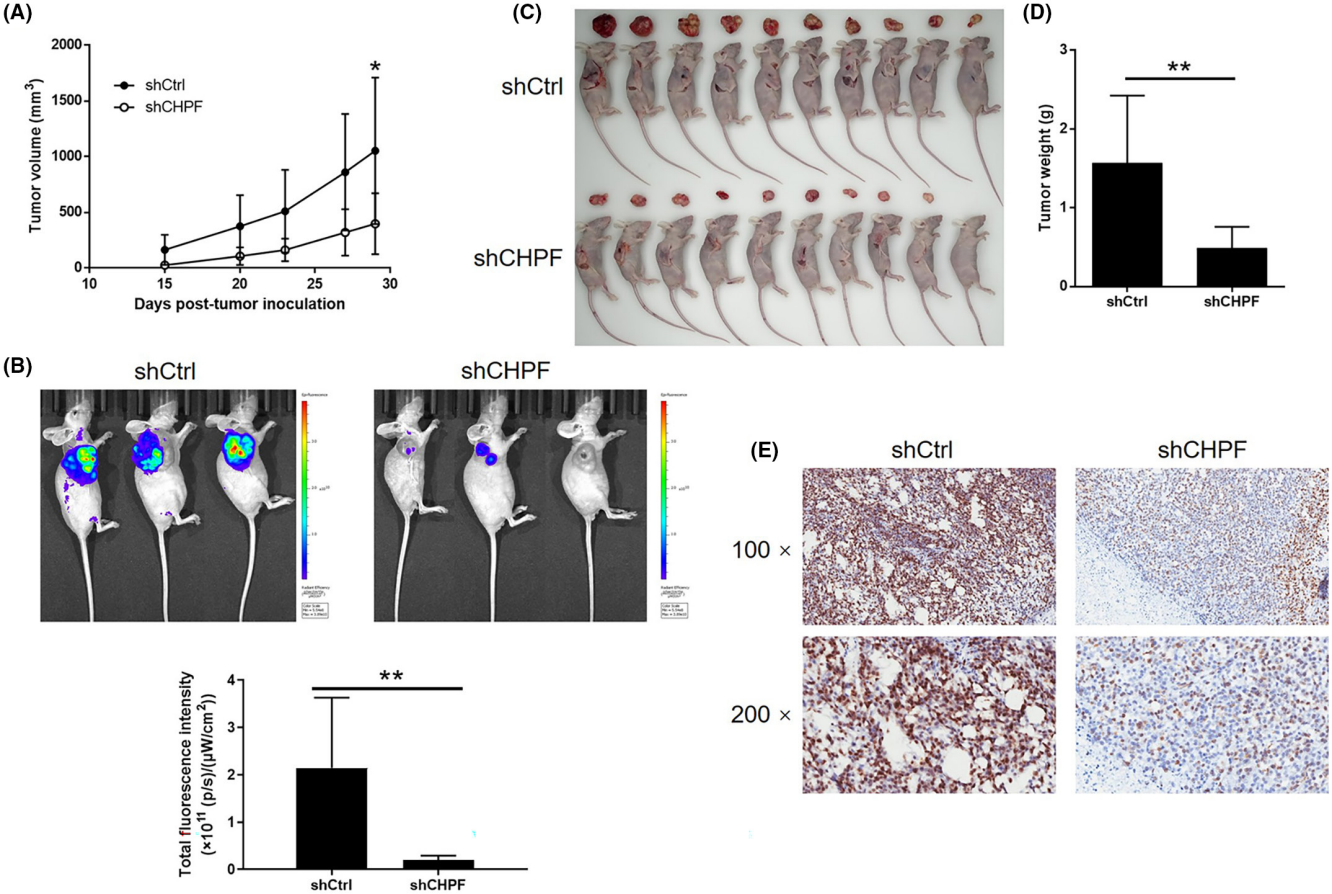
CHPF knockdown inhibited development of CRC in vivo. Mice xenograft models were constructed by subcutaneously injecting RKO cells with or without CHPF knockdown. (A) The volume of tumours in mice was measured and calculated at indicated intervals during the culture of the animal models. (B) In vivo imaging of the tumours on mice was facilitated by the injection of D‐luciferin before sacrificing the mice. (C) The photos of the tumours removed from the sacrificed mice were collected by a digital camera. (D) Tumour weight was measured after obtaining the removed tumours from animal models. (E) Tumour sections obtained from the removed tumours were subjected to IHC analysis to detect the expression of Ki‐67. The data were expressed as mean ± SD (*n* ≥ 3), *p < 0.05; **p < 0.01; ***p < 0.001.

To evaluate the influence of CHPF on colorectal cancer (CRC) development in vivo, xenograft mouse models were established by injecting RKO cells with or without CHPF knockdown. Tumour volume was monitored over time while the mice were housed in their cages. Notably, the shCHPF group displayed significantly slower tumour growth compared to the control group (*p* < 0.05, Figure 3A). In vivo imaging was performed prior to sacrificing the mice to visualize tumour development in situ. The fluorescence intensity was notably weaker in the shCHPF group, and the tumours in this group were significantly smaller (*p* < 0.05, Figure 3B). Upon sacrificing the mice, photographs of the tumours were taken (Figure 3C), and the tumours were weighed. The data unequivocally demonstrated that the shCHPF group had significantly smaller tumours (*p* < 0.01, Figure 3D). Furthermore, the Ki‐67 index, a proliferation marker, was determined through immunohistochemistry (IHC) analysis of histological sections from the tumours in each group. The shCHPF group exhibited a significantly lower Ki‐67 index compared to the shCtrl group (Figure 3E). These findings collectively indicate that CHPF knockdown suppresses the growth of CRC tumours in vivo.

### 3.4 Investigating the downstream mechanisms associated with the CHPF‐induced regulation of CRC

**FIGURE 4:**
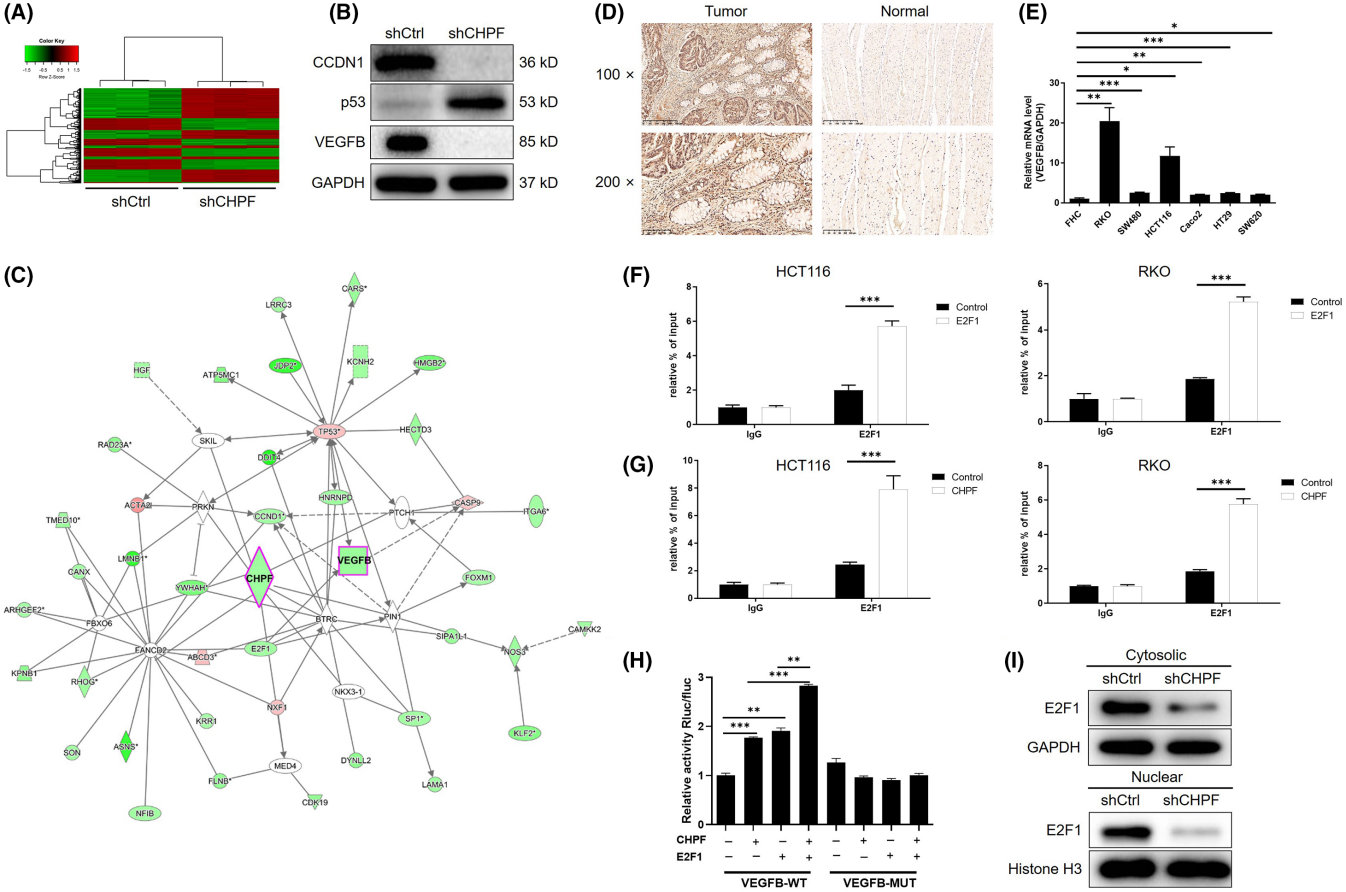
CHPF knockdown may inhibit CRC through regulation of VEGFB. (A) Heatmap of the RNA sequencing performed on RKO cells with or without CHPF knockdown (3 v 3). (B) The expression of Cyclin D1, p53 and VEGFB in the CHPF knockdown cells was verified by western blotting. (C) The CHPF‐related interaction network was predicted based on IPA analysis. (D) The expression of VEGFB in CRC tumour tissues and normal tissues was detected and compared by IHC analysis. (E) The background expression of VEGFB in normal human epithelial cell line FHC and CRC cell lines including HT‐29, SW480, SW620, RKO, HCT116 and Caco2 was detected by qPCR. (F, G) E2F1 binding to the promoter of VEGFB, which could be enhanced by E2F1 or CHPF overexpression, was detected by ChIP‐qPCR in RKO cells. (H) RKO cells were co‐transfected with VEGFB promoter luciferase reporter (wild type WT or mutated MUT) and CHPF or E2F1 overexpression plasmids followed by analysis of luciferase activity. (I) The cytosolic and nuclear expression of E2F1 was detected by western blotting in HCT116 cells with or without CHPF knockdown. The data were expressed as mean ± SD (*n* ≥ 3), *p < 0.05; **p < 0.01; ***p < 0.001.

Subsequently, a gene chip analysis was conducted to examine the gene expression profile in RKO cells with or without CHPF knockdown (3 v 3). In total, 502 differentially expressed genes (DEGs) were identified, with 301 upregulated and 201 downregulated genes (Figure 4A, Figure S4 and Table S3). Further analysis using IPA revealed significant enrichment of the p53 signalling pathway among the identified DEGs, consistent with the downregulation of p53 expression following CHPF knockdown (Figure S4). Enrichment in the VEGF signalling pathway was also observed (Figure S4). Moreover, analysis of the IPA disease and function database highlighted cancer as the most relevant disease regulated by CHPF (Figure S4). To validate these findings, a subset of DEGs was selected for verification using qPCR (Figure S4) and western blotting (Figure 4B). Integrating the bioinformatics data with our analysis of the CHPF‐related interaction network, we proposed that CHPF may exert its functional role in CRC through the regulation of VEGFB, one of the most downregulated DEGs (Figure 4C). To confirm this hypothesis, the expression of VEGFB was assessed in CRC tissues and normal tissues, revealing a significant downregulation of VEGFB in CRC (Figure 4D). Additionally, the expression of VEGFB in CRC cell lines was examined using qPCR, demonstrating higher expression levels in CRC cell lines compared to normal cells (Figure 4E). Based on the prediction of E2F1 as a transcriptional factor of VEGFB using the Cistrome Data Browser,^27^ chromatin immunoprecipitation (ChIP)‐qPCR was performed on HCT116 and RKO cells with or without E2F1 overexpression or CHPF overexpression. The results clearly demonstrated the interaction between E2F1 and the VEGFB promoter (Figure 4F). Furthermore, CHPF overexpression significantly enhanced the interaction between E2F1 and the VEGFB promoter (Figure 4G). Moreover, the regulatory mechanism involving CHPF‐induced VEGFB expression through E2F1‐mediated transcription was confirmed using luciferase assays, which indicated the enhancement or suppression of E2F1‐mediated transcription of VEGFB by CHPF overexpression or knockdown (Figure 4H and Figure S5). Additionally, CHPF knockdown downregulated the expression of E2F1 in both the cytoplasm and nucleus, potentially influencing the E2F1‐mediated transcriptional regulation of VEGFB (Figure 4I). Taken together, these results suggest that VEGFB may represent a potential downstream target of CHPF in the regulation of CRC.

### 3.5 VEGFB knockdown attenuated the effects of CHPF overexpression in CRC

**FIGURE 5:**
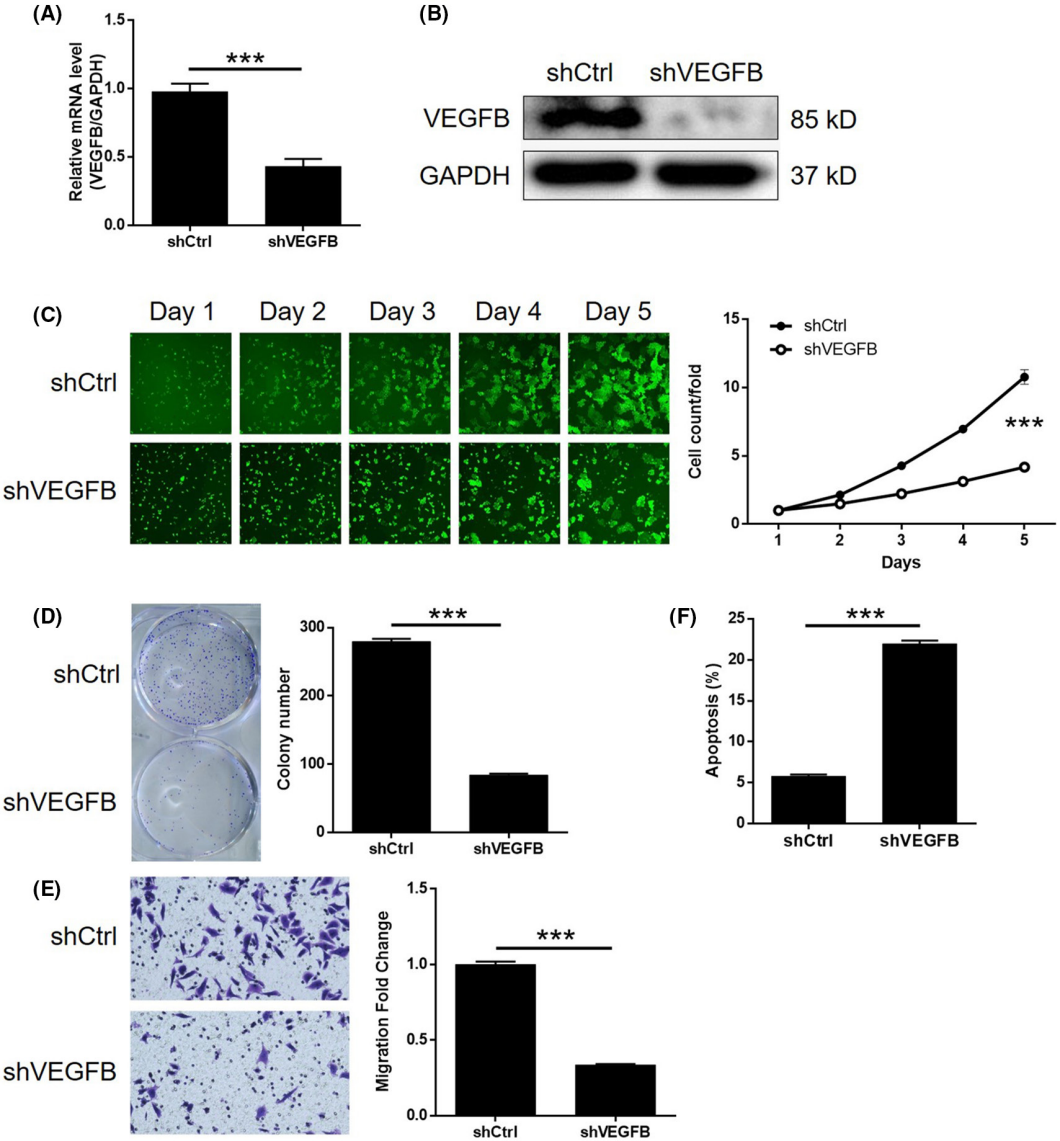
Knockdown of VEGFB inhibited development of CRC in vitro. (A) The efficiency of VEGFB knockdown in Caco2 cells was detected by qPCR. (B) The knockdown of VEGFB in Caco2 cells was confirmed by western blotting. (C) The regulation of cell proliferation by VEGFB knockdown in Caco2 cells was evaluated by Celigo cell counting assay. (D) The influence of colony formation ability of Caco2 cells by VEGFB knockdown was examined by colony formation assay. (E) The effects of VEGFB knockdown on cell migration of Caco2 cells were assessed by Transwell assay. (F) Flow cytometry was performed to detect cell apoptosis of Caco2 cells with or without VEGFB knockdown. The figures are representative data from at least three independent experiments. The data were expressed as mean ± SD (*n* ≥ 3), *p < 0.05; **p < 0.01; ***p < 0.001.

**FIGURE 6:**
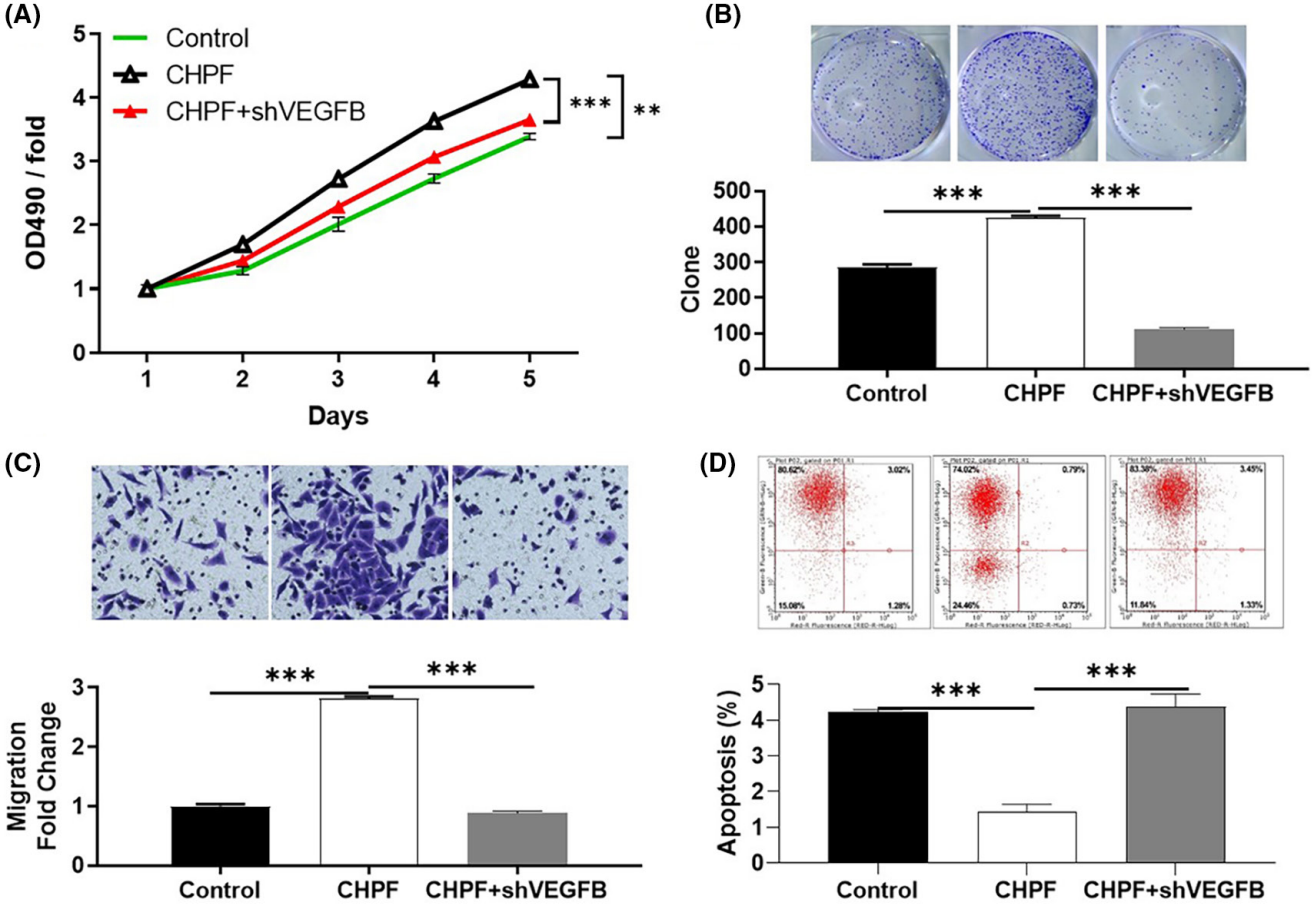
VEGFB knockdown alleviated the effects of CHPF overexpression on CRC. The results of MTT assay (A), colony formation assay (B), Transwell assay (C) and flow cytometry (D) showed that the effects of CHPF overexpression on cell proliferation, colony formation, cell migration and cell apoptosis could be alleviated by VEGFB knockdown. The figures are representative data from at least three independent experiments. The data were expressed as mean ± SD (*n* ≥ 3), *p < 0.05; **p < 0.01; ***p < 0.001.

To investigate the potential synergistic effect of CHPF and VEGFB on CRC development, Caco2 cells with low CHPF expression and high VEGFB expression were generated and validated. Three experimental groups were established: the CHPF overexpression group (CHPF group), the VEGFB knockdown group (shVEGFB group) and the simultaneous CHPF overexpression and VEGFB knockdown group (CHPF+shVEGFB group). After confirming VEGFB knockdown through qPCR (55.96% knockdown) and western blotting (*p* < 0.001, Figure 5A,B), various cellular functions were investigated. In the shVEGFB group, a significant reduction in cell proliferation (61.17%), colony formation (69.96%) and cell migration (66.33%) was observed (*p* < 0.001, Figure 5C–E). Additionally, there was a 3.79‐fold increase in the proportion of apoptotic cells (*p* < 0.001, Figure 5F), suggesting that VEGFB and CHPF may have similar roles in CRC. On the other hand, CHPF overexpression resulted in a significant increase in cell proliferation, colony formation and cell migration (*p* < 0.001, Figure 6A–C), while suppressing cell apoptosis (Figure 6D). Importantly, it was discovered that the enhanced malignant phenotypes induced by CHPF overexpression could be significantly alleviated by VEGFB knockdown (Figure 6), highlighting the crucial role of VEGFB in CHPF‐mediated regulation of CRC.

## 4 DISCUSSION

CS is widely distributed in diverse tissues^15^ and plays vital functions in the development of brain neural networks, inflammatory responses, immune defence against infections, cell proliferation and tissue architecture.^28^ Furthermore, it exhibits physiological properties, including the suppression of axonal regrowth after spinal cord injury^29^ and the prevention of aberrant myocardial remodelling.^30^ Additionally, cancer has also been identified as a key disease regulated by CS. For example, previous studies have demonstrated the inhibitory effects of shark chondroitin sulfate (CS) on liver cancer, its ability to induce apoptosis in multiple myeloma and breast cancer cells, as well as its inhibitory impact on tumour growth in mouse models of breast cancer.^31^ CHPF, an indispensable glycosyltransferase involved in chondroitin sulfate (CS) biosynthesis, serves as a crucial cofactor in the production of the repetitive disaccharide motif within CS molecules.^15^ Given the diverse physiological roles of chondroitin sulphate (CS), it is reasonable to hypothesize that the glycosyltransferase CHPF plays a significant role in modulating cellular processes such as cell division, differentiation, organismal development and disease pathogenesis. Moreover, emerging evidence has elucidated the association between CHPF and multiple malignancies. Notably, a study by Hou et al. unveiled the inhibitory effect of CHPF knockdown on the advancement of lung adenocarcinoma, both in experimental models and in clinical specimens.^32^ In their investigation, the researchers additionally noted an elevation in CHPF expression in non‐small cell lung cancer (NSCLC) and underscored the suppressive consequences of CHPF suppression on cellular proliferation, apoptosis and cell cycle progression. These findings provide compelling evidence for considering CHPF as a promising therapeutic target for NSCLC.^33^ Likewise, a study conducted by Ye et al. revealed the role of CHPF as an oncogenic factor in oesophageal squamous cell carcinoma, establishing its prognostic significance.^34^ More importantly, a recent study emphasized that CHPF is able to regulate the expression of E2F1 through UBE2T‐mediated ubiquitination, thus promoting gastric cancer development.^35^ However, the precise functional implications of CHPF in colorectal cancer (CRC) development have yet to be elucidated, as there is currently limited knowledge in this area.

Both the outcomes of our study and the data shown by TCGA database showed that the upregulation of CHPF in CRC, as well as the potential involvement of CHPF in CRC progression. The promotion of CRC by CHPF also could be observed through loss‐of‐function study based on CHPF knockdown CRC cell models and gain‐of‐function study based on CHPF overexpression CRC cell model. It was demonstrated that CHPF may regulate CRC progression through influencing cell proliferation, colony formation and cell migration. Not surprisingly, the in vivo growth of xenografts formed by CHPF knockdown cells was also inhibited. These results suggest that CHPF plays a role as a cancer promoter in the progression of CRC, as its role is similar in NSCLC and ESCC.

Angiogenesis plays a crucial role in the proliferation and dissemination of cancer cells, with the vascular endothelial growth factor (VEGF) family exerting significant control over this process.^36^, ^37^ Till now, research aiming at uncovering the functions of VEGFB, one of the 7 members includes VEGFA, VEGFB, VEGFC, VEGFD, VEGFE, VEGFF and placenta growth factor (PlGF) in VEGF family, is still relatively rarely seen.^38^, ^39^, ^40^ Except for the role in angiogenesis, VEGFB was reported to play important role in the protection of coronary vessels and myocardium.^41^ The expression of VEGFB in mice myocardial models with myocardial infarction or heart failure induced by aortic coarctation was both significantly downregulated.^41^ Moreover, studies have shown that the expression of VEGFB is abundant in embryonic and adult muscle tissues, and it could promote endothelial cell proliferation and local vascular growth, which is conducive to the growth and metastasis of malignant tumours.^42^ It was deduced that the expression of VEGFB in ovarian cancer, colorectal cancer, renal cancer and prostate cancer was significantly upregulated, so it may play a role in the initial stage of tumorigenesis.^43^ Furthermore, Yang et al. indicated that, as a marker of poor prognosis in cancer patients, targeting VEGFB may be an important treatment strategy for cancer metastasis.^42^

Herein, we identified VEGFB as a potential downstream target of CHPF because of its co‐expression pattern with CHPF. It was also demonstrated that CHPF may enhance the expression of VEGFB through E2F1‐mediated transcriptional regulation. In agreement with the general tumour‐promoting profile of VEGFB, its upregulation in CRC tissues and regulatory functions in CRC progression were also displayed in this study. More importantly, it was illustrated that the CHPF overexpression‐induced CRC promotion is dependent on the expression of VEGFB to some extent, suggesting that VEGFB is a key aspect in the downstream pathway of CHPF in CRC.

Collectively, although there are still some drawbacks of this study, such as the limited number of clinical specimens and the blurry involvement of angiogenesis in CHPF‐induced regulation of CRC, we can come to a conclusion that CHPF has been identified as a crucial factor in the progression of CRC, which possesses the potential application in the targeted therapy of CRC.

## AUTHOR CONTRIBUTIONS

**Yuan Huang:** Conceptualization (equal); methodology (equal); writing – original draft (equal); writing – review and editing (equal). **Zhen Zhang:** Conceptualization (equal); data curation (equal); methodology (equal); software (equal). **Hanxing Tong:** Methodology (equal); resources (equal); software (equal). **Wenzheng Qin:** Conceptualization (equal); investigation (equal); software (equal). **Quanlin Li:** Data curation (equal); formal analysis (equal). **Lili Ma:** Formal analysis (equal); investigation (equal). **Zhong Ren:** Investigation (equal); methodology (equal). **Weifeng Chen:** Formal analysis (equal); validation (equal). **Yiqun Zhang:** Resources (equal); software (equal). **Yunshi Zhong:** Investigation (equal); resources (equal). **Liqing Yao:** Investigation (equal); methodology (equal). **Pinghong Zhou:** Conceptualization (equal); funding acquisition (equal); methodology (equal); project administration (equal); writing – original draft (equal); writing – review and editing (equal).

## FUNDING INFORMATION

This work was financially supported by the Shanghai Engineering and Research Center of Diagnostic and Therapeutic Endoscopy (Nos. 16DZ2280900 and 19DZ2280100).

## CONFLICT OF INTEREST STATEMENT

The authors declare no conflict of interest.

## Supporting information


Figure S1.

Figure S2.

Figure S3.

Figure S4.

Figure S5.

Table S1.

Table S2.

Table S3.

## Data Availability

All data generated or analysed during this study are included in this published article and its supplementary information files.
