# Supplementary figures and images for: Chondroitin polymerizing factor promotes development and progression of colorectal cancer via facilitating transcription of VEGFB
Source: J Cell Mol Med. 2024 May 22;28(10):e18268. doi: 10.1111/jcmm.18268 (PMC11109815; doi:10.1111/jcmm.18268)

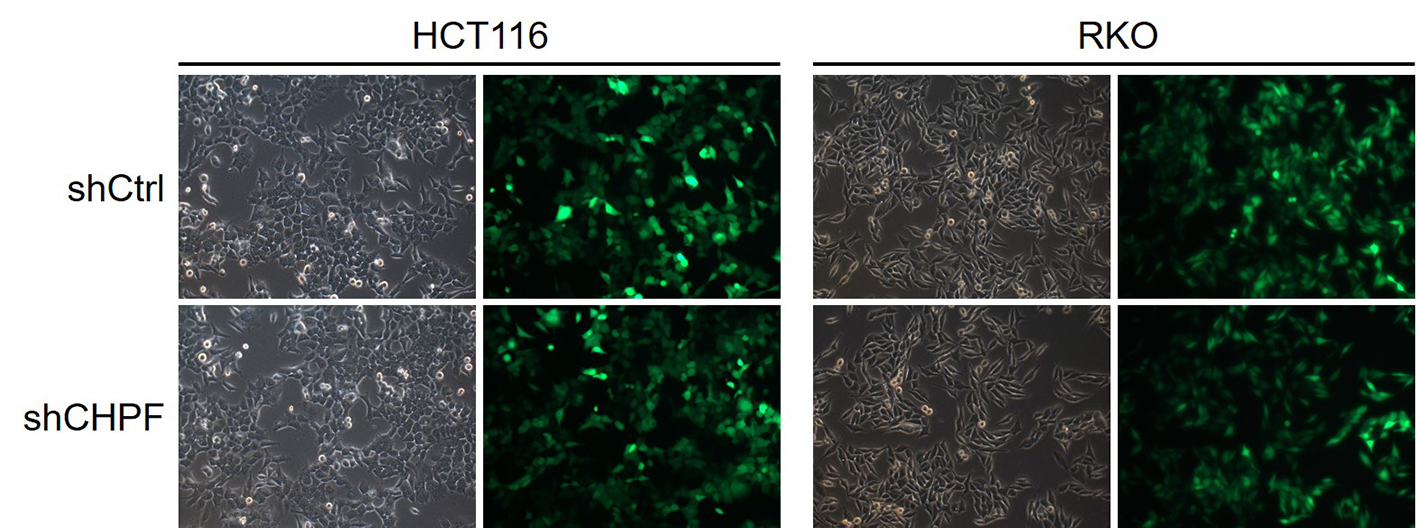

Supplement: Supplementary file 1 — Figure S1. [file JCMM-28-e18268-s004.tif]

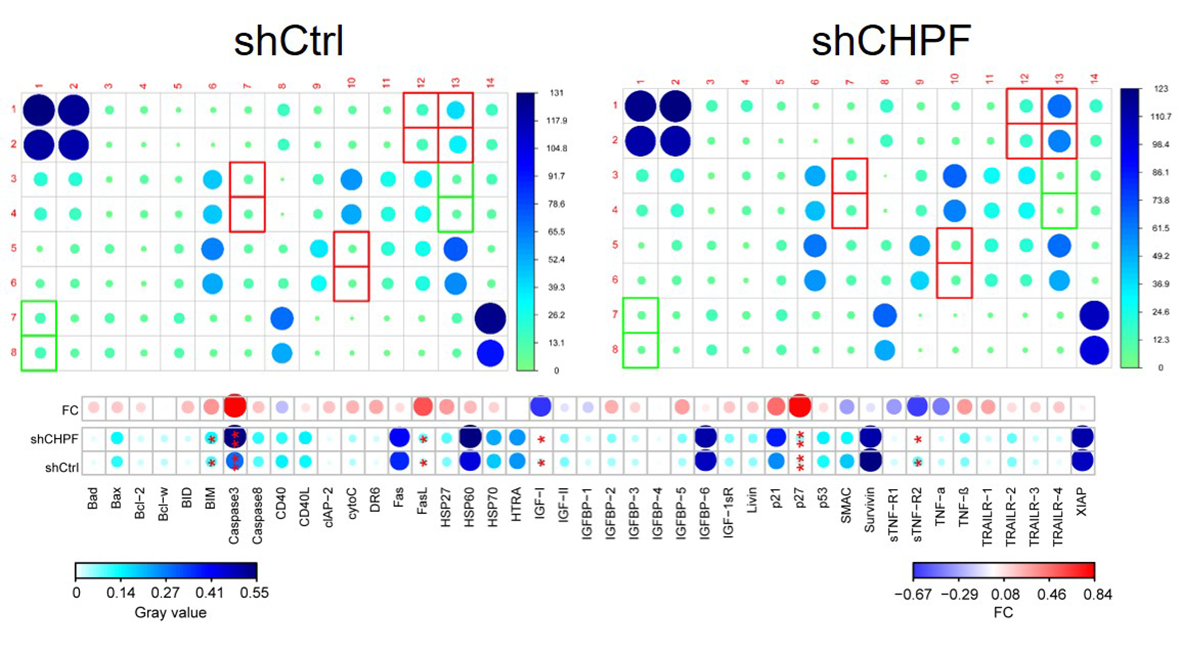

Supplement: Supplementary file 2 — Figure S2. [file JCMM-28-e18268-s005.tif]

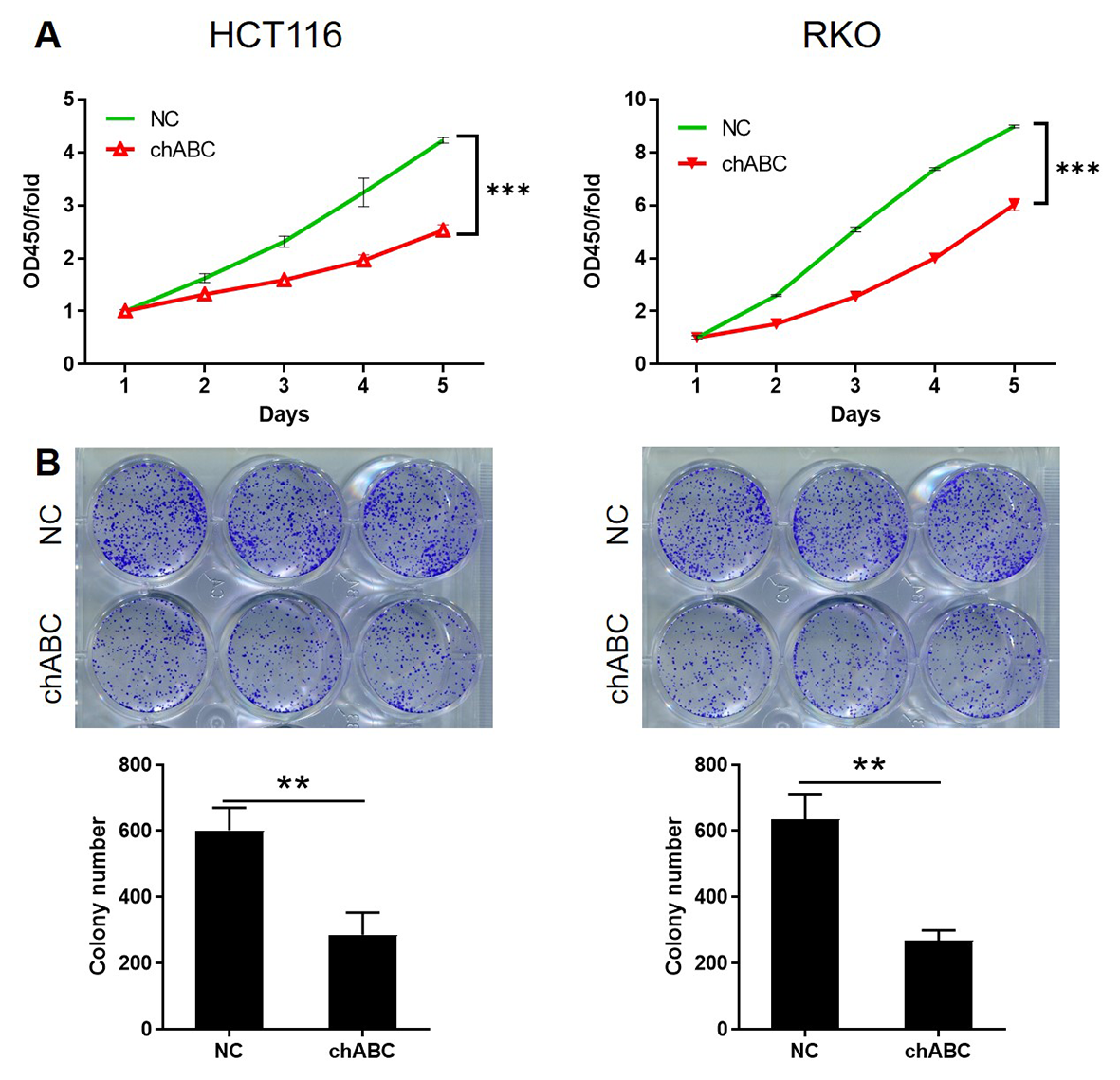

Supplement: Supplementary file 3 — Figure S3. [file JCMM-28-e18268-s006.tif]

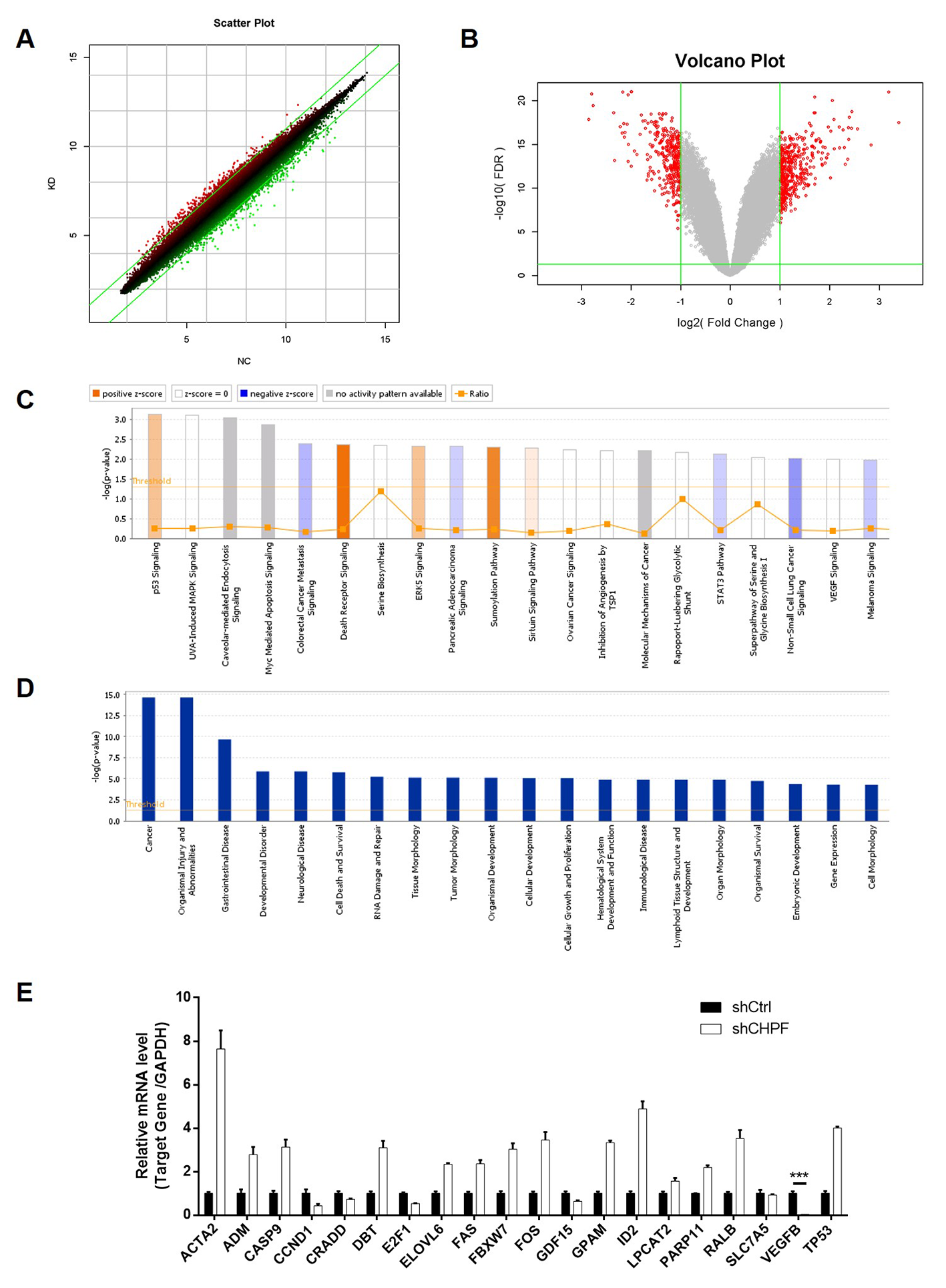

Supplement: Supplementary file 4 — Figure S4. [file JCMM-28-e18268-s007.tif]

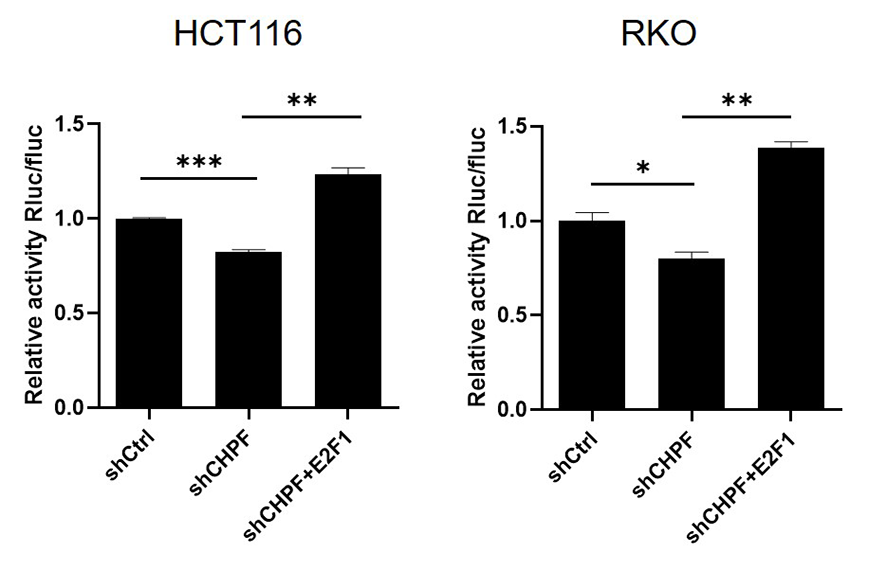

Supplement: Supplementary file 5 — Figure S5. [file JCMM-28-e18268-s001.tif]
