# Supplementary material for: Chondroitin polymerizing factor promotes development and progression of colorectal cancer via facilitating transcription of VEGFB
Source: J Cell Mol Med. 2024 May 22;28(10):e18268. doi: 10.1111/jcmm.18268 (PMC11109815; doi:10.1111/jcmm.18268)
Supplement: Supplementary file 6 — Table S1. [file JCMM-28-e18268-s003.docx]

Table S1 Antibodies used in western blotting and IHC

| Primary antibodies | Dilution in WB | Source species | Company | Catalog No. |
| --- | --- | --- | --- | --- |
| CHPF | 1:1000 | Rabbit | Abcam | ab224495 |
| GAPDH | 1:3000 | Rabbit | Bioworld | AP0063 |
| VEGFB | 1:500 | Rabbit | Abcam | ab185696 |
| Cyclin D1 | 1:1000 | Rabbit | CST | 2978 |
| p53 | 1:1000 | Mouse | abcam | ab26 |
| DYKDDDDK Tag* | 1:50/1:1000 | Rabbit | CST | 14793 |
| HA | 1:25/1:3000 | Rabbit | abcam | ab9110 |
| * DYKDDDDK Tag binds to same epitope as Sigma's Anti-FLAG® M2 Antibody | | | | |
| Primary antibodies | Dilution in IHC | Source species | Company | Catalog No. |
| CHPF | 1:200 | Rabbit | Abcam | ab224495 |
| Ki-67 | 1:200 | Rabbit | Abcam | ab16667 |
| VEGFB | 1:200 | Rabbit | Abcam | ab185696 |
|  |  |  |  |  |
|  |  |  |  |  |
| Secondary antibody | Dilution |  | Company | Catalog No. |
| HRP Goat Anti-Rabbit IgG (WB) | 1:3000 |  | Beyotime | A0208 |
| HRP Goat Anti-Mouse IgG (WB) | 1:3000 |  | Beyotime | A0216 |
| HRP Goat Anti-Rabbit IgG (IHC) | 1:200 |  | Abcam | Ab111909 |
