# Supplementary material for: Chondroitin polymerizing factor promotes development and progression of colorectal cancer via facilitating transcription of VEGFB
Source: J Cell Mol Med. 2024 May 22;28(10):e18268. doi: 10.1111/jcmm.18268 (PMC11109815; doi:10.1111/jcmm.18268)
Supplement: Supplementary file 7 — Table S2. [file JCMM-28-e18268-s008.docx]

Table S2 Primers used in qPCR

| Gene | Forward primer sequence (5’-3’) | Reverse primer sequence (5’-3’) |
| --- | --- | --- |
| CHPF | GGAACGCACGTACCAGGAG | CGGGATGGTGCTGGAATACC |
| VEGFB | AAAGGACAGTGCTGTGAAGCC | TGGAGTGGGATGGGTGATGT |
| GAPDH | TGACTTCAACAGCGACACCCA | CACCCTGTTGCTGTAGCCAAA |
